# Erosive Arthritis and Hepatic Granuloma Formation Induced by Peptidoglycan Polysaccharide in Rats Is Aggravated by Prasugrel Treatment

**DOI:** 10.1371/journal.pone.0069093

**Published:** 2013-07-04

**Authors:** Analia E. Garcia, Mario C. Rico, Elisabetta Liverani, Raul A. DeLa Cadena, Paul F. Bray, Satya P. Kunapuli

**Affiliations:** 1 Sol Sherry Thrombosis Research Center, Temple University School of Medicine, Temple University Hospital, Philadelphia, Pennsylvania, United States of America; 2 Department of Physiology, Temple University School of Medicine, Temple University Hospital, Philadelphia, Pennsylvania, United States of America; 3 Department of Pharmacology, Temple University School of Medicine, Temple University Hospital, Philadelphia, Pennsylvania, United States of America; 4 Cardeza Foundation for Hematologic Research, Thomas Jefferson University, Philadelphia Temple University School of Medicine, Temple University Hospital, Philadelphia, Pennsylvania, United States of America; University Hospital Jena, Germany

## Abstract

Administration of the thienopyridine P2Y_12_ receptor antagonist, clopidogrel, increased the erosive arthritis induced by peptidoglycan polysaccharide (PG-PS) in rats or by injection of the arthritogenic K/BxN serum in mice. To determine if the detrimental effects are caused exclusively by clopidogrel, we evaluated prasugrel, a third-generation thienopyridine pro-drug, that contrary to clopidogrel is mostly metabolized into its active metabolite in the intestine. Prasugrel effects were examined on the PG-PS-induced arthritis rat model. Erosive arthritis was induced in Lewis rats followed by treatment with prasugrel for 21 days. Prasugrel treated arthritic animals showed a significant increase in the inflammatory response, compared with untreated arthritic rats, in terms of augmented macroscopic joint diameter associated with significant signs of inflammation, histomorphometric measurements of the hind joints and elevated platelet number. Moreover, fibrosis at the pannus, assessed by immunofluorescence of connective tissue growth factor, was increased in arthritic rats treated with prasugrel. In addition to the arthritic manifestations, hepatomegaly, liver granulomas and giant cell formation were observed after PG-PS induction and even more after prasugrel exposure. Cytokine plasma levels of IL-1 beta, IL-6, MIP1 alpha, MCP1, IL-17 and RANTES were increased in arthritis-induced animals. IL-10 plasma levels were significantly decreased in animals treated with prasugrel. Overall, prasugrel enhances inflammation in joints and liver of this animal model. Since prasugrel metabolites inhibit neutrophil function *ex-vivo* and the effects of both clopidogrel and prasugrel metabolites on platelets are identical, we conclude that the thienopyridines metabolites might exert non-platelet effects on other immune cells to aggravate inflammation.

## Introduction

For decades, decreasing platelet activation has been a target in the prevention of coronary artery disease [1] and an array of drugs have been discovered to selectively interfere with platelet function. Among those are prasugrel and clopidogrel, thienopyridine compounds that antagonized the P2Y_12_ receptor in platelets. Prasugrel was approved by the Food and Drug Administration of the United States to be used to reduce risk of thrombus formation on patients undergoing for percutaneous coronary intervention. However, prasugrel, a pro-drug that is considered more potent than clopidogrel, has been associated with an increased risk of primarily gastrointestinal bleeding [1]. Unlike other thienopyridine drugs, prasugrel is more easily metabolized than clopidogrel into its active and inactive metabolites, requiring fewer steps in the liver. The active metabolite binds covalently and irreversibly to the P2Y_12_ receptor [2], [3] and inhibits platelets more rapidly, potently and consistently than clopidogrel. Recently, clopidogrel response variability and drug resistance among patients has been reported due to the loss-of-function allele CYP2C19*2 [4]. In contrast, prasugrel treatment has not being implicated with any polymorphism and has shown higher efficacy than high-dose clopidogrel [5].

Increasing reports of development of arthritis, in particular acute migratory polyarthritis after clopidogrel regimen, raise the need for alternative treatments [6]–[13]. Prasugrel has not being implicated in these arthritogenic events, although prasugrel is a recent drug that has narrower clinical indications than clopidogrel. Koshal and co-workers [9] proposed prasugrel as an alternative anti-thrombotic drug to reduce the risk of drug-induced-arthritis. Platelets and platelet micro particles have been implicated in the perpetuation of arthritis, as shown by Lee and co-workers [14], who demonstrated aggravation of arthritis in the presence of micro particles from platelets. In their murine model, they demonstrated that the administration of serum from K/BxN animals provoked erosive arthritis in C57BL/6J mice and the daily administration of clopidogrel resulted in increased joint diameter and inflammation [14]. Comparable results were observed in a rat model of erosive arthritis induced by peptidoglycan polysaccharide PG-PS after treatment with clopidogrel [15], which raise concerns of the use of clopidogrel in patients with rheumatoid arthritis (RA). The ultimate mechanism of the detrimental effect of clopidogrel treatment observed in these two animal models is still unknown. Therefore, this study is intended to evaluate prasugrel, a thienopyridine pro-drug, in inflammation and to further investigate how these drugs affect inflammatory processes.

## Materials and Methods

### Erosive Arthritis Induction and Assessment

Thirty-two female pathogen-free Lewis rats obtained from Charles River Laboratories (Raleigh, NC), weighing between 160 - 180 grams, were randomly separated into the experimental groups and studied for 21 days. Sixteen animals received a single dose of 15 µg of rhamnose/gram of mean body weight of purified, sterile, PG-PS polymer (BD Lee Laboratories, Grayson, GA), administered by intraperitoneal (*i.p.)* injection on day 0. Of those animals, 8 animals, the PG-PS+prasugrel group, received 3 mg/kg daily oral dose of prasugrel (Effient® 10 mg tablets, prasugrel hydrochloride dissolved in 0.5% carboxyl methylcellulose as vehicle solution (Sigma-Aldrich Chemicals, St. Louis, MO), and the other 8, the PG-PS group, received only the vehicle solution. The other 16 animals received no PG-PS, 8 received oral daily dose of prasugrel and 8 received oral daily vehicle solution. Animals were weighed and examined daily. Under anaesthesia (EZ 1500, anaesthesia System from Euthanex Corp., for isofluorane [VetOne Pharmaceuticals]), ankle diameter was measured and arthritis severity was assessed as previously described [15], [16]. At day 21, rats were anesthetized, and blood samples were collected by cardiac puncture for hematology, plasma separation and for serum collection for chemical analyses. A 10∶1 ratio of blood in 3.8% sodium citrate was used as anticoagulant. The experimental protocol used in this study was fully approved by The Institutional Animal Care and Use Committee of Temple University School of Medicine.

### Hematology, Platelet and Blood Chemistry Studies

Fifty microliters of anticoagulated blood was used to carry out a detailed hematologic profile from each animal, utilizing the Hemavet® Multispecies Hematology System (Drew Scientific, Inc. Oxford, CT). Then, total blood was centrifuged in polypropylene tubes at 22°C at 100×g for 10 min to obtain platelet rich plasma (PRP). PRP was centrifuged again at 400×g for 10 min. Platelet-poor plasma was recovered and aliquots of supernatant were stored at −70°C for cytokine profile studies. Platelet pellets after centrifugation were resuspended in Tyrodes buffer (pH 7.4) containing 0.05 units/mL apyrase. Platelets were counted using the Hemavet® System. Aggregation was measured using light transmission under stirring conditions (900 rpm) at 37°C (P.I.C.A. Lumiaggregometer [Chrono-log Corp., Havertown, PA]). Aggregation tracings of 0.5 mL of washed platelets from each animal were recorded.

Serum from each animal was analyzed for chemistry profile including cholesterol, triglycerides, alanine aminotransferase (ALT), aspartate aminotransferase (AST), alkaline phosphatase, total bilirubin, glucose, phosphate, total protein, blood urea nitrogen (BUN), creatinine, albumin, calcium, sodium, potassium, and chloride levels (Charles River Laboratories, MA).

### Histopathology and Immunofluorescence

Hind paws were prepared as previously described [15]–[17]. In brief, samples were fixed, decalcified, embedded in paraffin, sectioned and stained with hematoxylin and eosin for microscopy. The degree of inflammation and articular injury was measured by a score system previously described in detail [15], [18]–[20]. Pathologic parameters were measured for synovial hyperplasia, cell infiltration, neovascular formation, fibrosis and pannus formation. The histopathological score ranged from 0 to 24.

Since in addition to joint inflammation, *i.p.* PG-PS-injection develops hepatomegaly and granuloma formation, liver was weighed and the percentage of the organ from the total body weight was calculated and plotted. Liver granuloma formation was assessed and measured using Bioquant^®^ software as previously described [16]. In brief, a percentage of the granuloma area over the total tissue area was calculated by tracing the perimeter of each granuloma. Several histological sections of the different groups were analyzed and plotted.

To further evaluate fibrosis in the affected joint tissue, we measured connective tissue growth factor (CTGF) protein levels by immunofluorescence, using previously described methods [16]. In brief, slides from the rat groups were deparaffinized, rehydrated and blocked with 10% BSA in HBSS buffer solution, followed by overnight incubation with monoclonal CTGF antibody (sc-101586, Santacruz Biotechnology Inc, CA). Slides were washed and then incubated with anti-mouse IgG-FITC labelled (sc-2010, Santacruz Biotechnology Inc, CA) for microscopy analysis. Control staining slides incubated with anti-mouse IgG conjugated isotype were used to evaluate non-specific binding. Immunofluorescence present in the tissue was quantified as a percentage of the area of CTGF immunoreactivity by Image J software.

### Plasma Cytokine Profiles

Plasma aliquots from each animal were utilized for detection of interleukin (IL)-1β, IL-4, IL-6, IL-10, IL-17, macrophage inflammatory protein (MIP)-1α; monocyte chemotactic protein (MCP)-1, tumor necrosis factor (TNF)-α and regulated upon activation, normally t-expressed and presumably secreted chemokine (RANTES) plasma levels by the Luminex® System (AssayGate, Inc. Ijamsville, MD). Plasma levels of platelet factor 4 (PF4) were measured by ELISA (AssayGate, Inc. Ijamsville, MD).

### Statistical Analysis

Differences among groups were statistically analyzed using one-way analysis of variances (ANOVA); Bonferroni’s multiple comparison test was used as post-test analyses. *P*<0.05 was considered to be significant. The joint diameter was analyzed as a continuous variable of all analyses. Hepatic granuloma area and giant cell number was analyzed by two-way unpaired *t-test.* Data are reported as mean ± standard error of the mean (SEM) for each group.

## Results

### PG-PS-induced Arthritis Animals Treated with Prasugrel Exhibit Greater Clinical Signs of Arthritis

**Figure 1:**
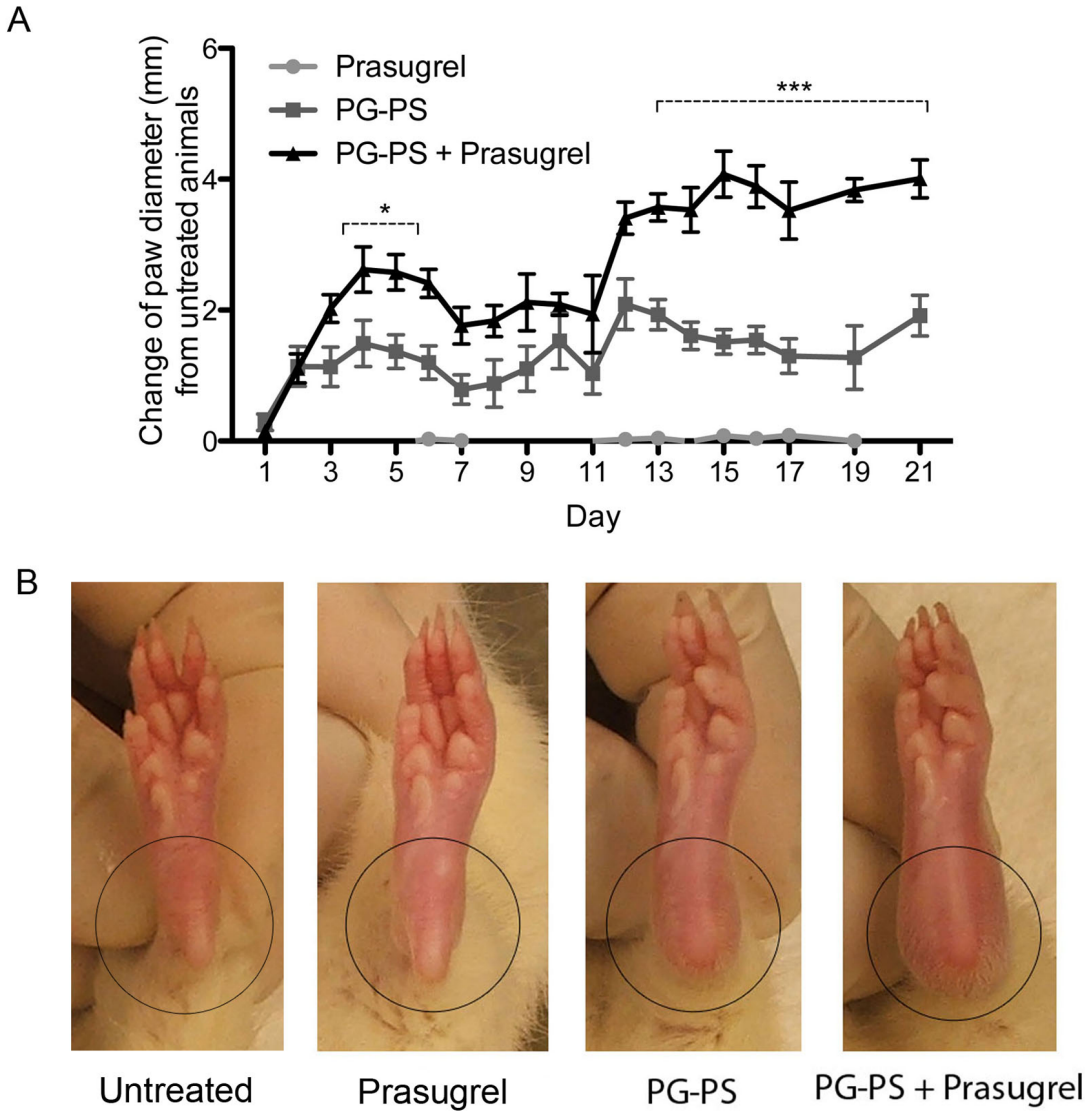
Prasugrel increases the clinical and pathological manifestations of PG-PS-induced arthritis. (**A**) Daily measurements of ankle joint diameter. Values represent the change of hind paw diameter in millimetres (n = 16 measurements [8 rats] per group). Values plotted are the mean ± SEM of the measurements. *p<0.05 and ***p<0.001 between PG-PS+prasugrel vs. PG-PS groups. (**B**) Macroscopic images of paw and ankle joint of the animals at day 21. Increase joint diameter associated with visible signs of inflammation.

To evaluate the effect of prasugrel during inflammation, PG-PS was administered to rats at the beginning of the study. Daily paw diameter was measured to evaluate and document the progression of the erosive arthritis. The paw diameter in the PG-PS arthritis-induced animals was significantly increased after the PG-PS injection following the typical biphasic pattern, acute and chronic phases, observed in this animal model (panel A of Fig. 1). From those animals, the addition of prasugrel showed greater increase in paw diameter during the acute phase (days 4 to 6, *p<0.05). Further increase in paw diameter was observed during the chronic phase with prasugrel treatment (days 13 to 21, ***p<0.001). It is important to mention that all PG-PS-induced arthritis animals reached a plateau in the diameter measurements at the beginning of the chronic phase. However, the joints of the prasugrel-treated animals were notably more inflamed and with visible signs of severe arthritis than the joints of the PG-PS animals (panel B of Fig. 1). Since we found no differences in any of the parameters evaluated between the untreated group and prasugrel alone group, hereafter we will refer those groups as control groups, unless specified otherwise.

### Histomorphometric and Immunofluoresce Analyses of the Limb Joints Confirmed the Macroscopic Findings

**Figure 2:**
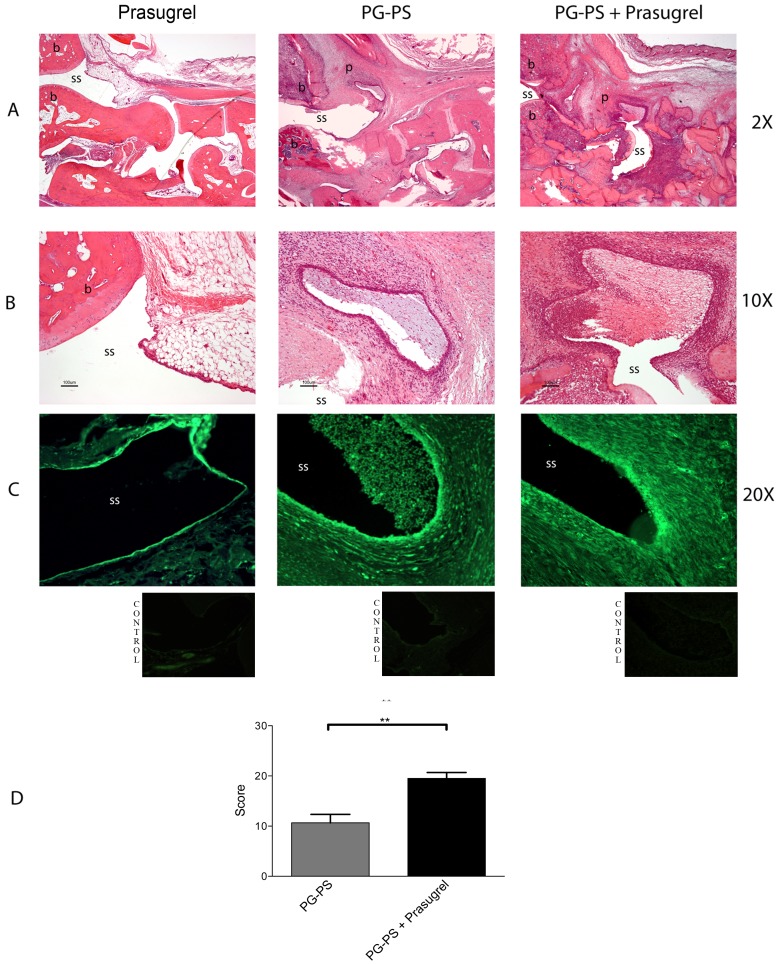
Differences observed in the PG-PS-induced erosive arthritis after prasugrel treatment. Histological examination of ankle joints of rats from different groups of sections stained with H&E at 2X and 10× magnifications (**A** and **B**, respectively). Sections from untreated animals (not shown) and prasugrel alone revealed no histopathological signs of inflammation. (**C**) CTGF expression was assessed with fluorescence microscopy (green – FITC). Images are representative of 4 different animals per group. Lower and right photographs underneath are representative of the control isotype stainings. (**D**) Histopathological score. Values plotted are the mean of the measurements ± SEM, (n = 4 per group) **p<0.01. b, bone; ss, synovial space; p, pannus.

Histology of the ankle joints of the prasugrel-treated animals revealed greater synoviocyte hyperplasia, leukocyte infiltration, fibrosis, bone destruction, and pannus formation than the joints of the PG-PS controls (panel A of Fig. 2). Photographs were taken from the histology slides at 2× magnification to show the tibio-tarsal articulation. In the joints of the control groups (untreated [not shown] and prasugrel alone groups), the synovial space remained intact and limited by the synovium of single lining of cells and intact bone surface. In contrast, joints of the PG-PS-induced arthritis animals showed signs of chronic inflammation: the synovium was covered with multiple layers of cells, the synovial space was invaded with pannus, the peri-synovial tissues were infiltrated with inflammatory cells, normal tissue became fibrotic and the bone was severely eroded and deformed. Greater detail is appreciated at 10× magnification of the tibio-tarsal joint (panel B of Fig. 2), where the severe synovial hyperplasia and the neovascular formation can be observed. Overall, in all the histology parameters evaluated, the severity of the arthritis was greater in the prasugrel treated animals.

The downstream protein of the transforming growth factor beta (TGF-ß) pathway, CTGF, is considered an excellent marker for fibrosis in cartilage and bone tissue. Tissue fibrosis assessed by immunofluoresce of CTGF was increased in arthritic rats treated with prasugrel. CTGF protein expression was significantly increased in the prasugrel treated arthritic animals compared to prasugrel alone control. Immunofluorescence levels of CTGF in the PG-PS control were 4.08±0.17 (% area CTGF immunoreactivity) and the PG-PS+prasugrel group values were 9.05±0.22 (% area CTGF immunoreactivity), ***p<0.001. Representative photographs of the immunofluorescence are shown in panel C of Fig. 2. Underneath of each photograph in panel C are representative small pictures of the isotype control stainings.

Measurements of the pathological changes in the joints were assessed and quantified as previously described [15]. The synoviocyte hyperplasia, measured as the predominant cell depth of the synovial lining, was 13±1.72 SEM in PG-PS control animals vs. 50.14±5.46 SEM in prasugrel treated animals. Proliferative blood vessels quantified as the number of vessels per high power field (HPF) was 7.5±0.34 SEM in PG-PS control animals vs. 17.83±3.75 SEM in prasugrel treated animals. The percentage of perivascular infiltrates of leukocytes was 50.89% ±2.6 SEM in PG-PS control animals vs. 92.29±2.82 SEM in prasugrel treated animals. The percentage of fibrotic tissue was 20.99±2.86 SEM in PG-PS control animals vs. 44.57±2.44 SEM in prasugrel treated animals. All of these parameters were summarized in the histopathological score, which as 10.67±1.66 SEM for PG-PS control animals while in the PG-PS+prasugrel treated animals was 19.5±1.18 SEM (**p<0.01) (Panel D of Fig. 2). These results are in accordance with the physical manifestations observed in the animals.

### Hepatomegaly with Granuloma Formation was Associated with PG-PS Induction and Increased with Prasugrel Treatment

**Figure 3:**
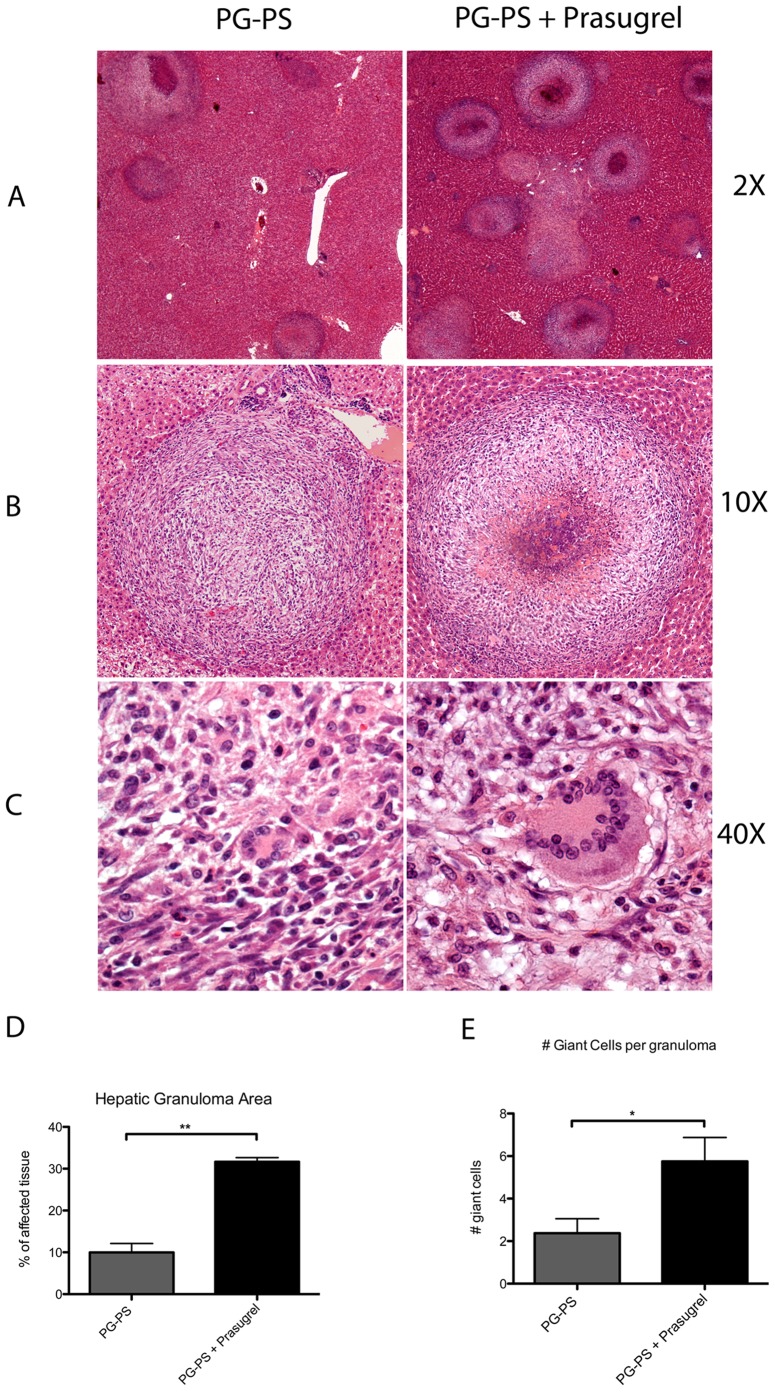
Increase in liver granuloma formation in the prasugrel treated PG-PS-induced animals. Photographs of liver tissue, stained with H&E, showing the formation of granulomas in the hepatic tissue. Upper panel photographs represent hepatic tissue at 2× magnification (**A**) and middle panel photographs are magnifications of granulomas (10X) (**B**) with the presence of giant cells (40X) (**C**). Images are representative of 4 animals per group. Percentage of granuloma area in the liver (**p<0.01) (**D**) and the presence of giant cells at the granuloma (*p<0.05) (**E**) were measured and plotted. Values represent the mean ± SEM, (n = 6).

Besides the arthritic manifestations, we evaluated the inflammatory effects of PG-PS and the addition of prasugrel treatment in liver. All animals treated with PG-PS showed an increase in organ weight of the liver (2-fold increase from control groups). Interestingly, not only was the liver granuloma area significantly increased in animals treated with prasugrel (3-fold increase in animals treated with prasugrel) (panels A, B and D of Fig. 3) but more specifically, the numbers of giant cells were also increased in prasugrel treated animals (2-fold increase in animals treated with prasugrel) (panels C and E of Fig. 3). Indeed the effect of prasugrel seems to aggravate liver pathology after PG-PS, and is not localized to the joints. No difference was noted in liver histology between healthy mice and prasugrel-treated healthy animals (data not shown).

### Neutrophil and Platelet Count are Increased in PG-PS-induced Arthritis Animals Treated with Prasugrel

**Figure 4:**
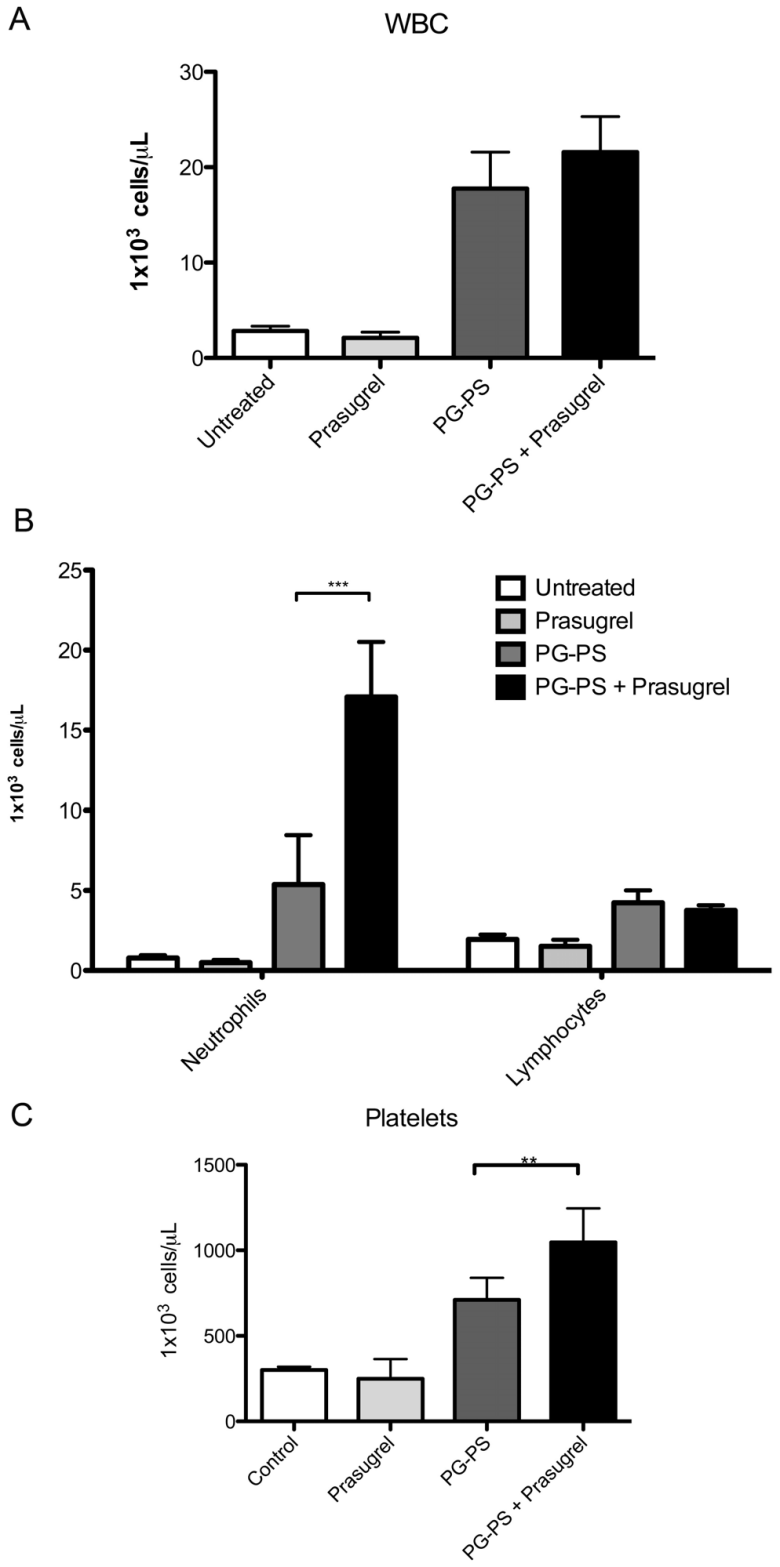
Hematology cell counts following prasugrel treatment in PG-PS- induced arthritis model. White blood cells (WBC) (**A**), differential (**B**) and platelet (**C**) blood counts at day 21. Cell counts are 10^3^/µL. Values are mean ± SEM, (n = 6), **p<0.01, ***p<0.001.

**Figure 5:**
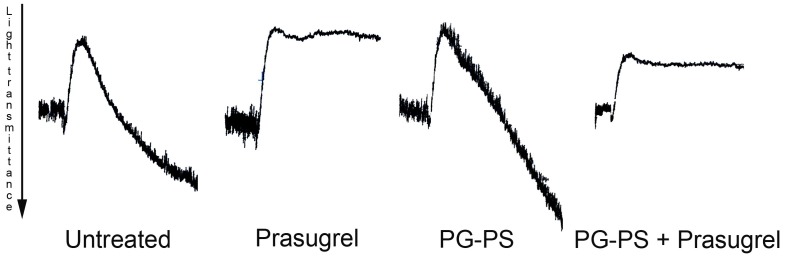
Platelet aggregation studies using 2MeSADP. Effect of 2MeSADP (100 nM) on platelet aggregation in washed platelets isolated from the blood of the rats. Traces are representative (n = 3) of each of the groups of the study.

Compared to untreated rats, prasugrel treatment alone did not alter the blood (Fig. 4, panels A–C). Both groups treated with PG-PS showed an increase in WBC and platelet counts (Fig. 4. panels A, C). The leukocytosis was driven primarily at the expense of the number of neutrophils and secondarily to lymphocytes (Fig. 4, panel B). Prasugrel treatment in arthritic animals resulted in greater neutrophilia (PG-PS: 4.8±0.57×10^3^ cells/µL, PG-PS+Prasugrel 10.4±6.6×10^3^ cells/µL, *p<0.05) (Fig. 4, panel B) and thrombocytosis (PG-PS: 710.8±52.65×10^3^ cells/µL vs. PG-PS+Prasugrel 1,047±89.03×10^3^ cells/µL, *p<0.05) (Fig. 4, panel C). Notably, Prasugrel had not effect on lymphocyte count in control or PG-PS treated rats. Platelet aggregation was inhibited after treatment with prasugrel, in animals treated with prasugrel alone and in animals treated with prasugrel and PG-PS-induced arthritis (Fig. 5).

### Proinflammatory Cytokines were Increased after PG-PS Arthritis Induction and Plasma Levels of Interleukin 10 were Decreased in the PG-PS-induced Arthritis Animals Treated with Prasugrel

The cytokine profile summarized in Table 1, showed an increase in pro-inflammatory cytokines in both treated and untreated PG-PS arthritis-induced animals, compared with control animals. Interestingly, a trend of increase is shown for PF4, MCP-1 and RANTES cytokines, confirming a greater increase in inflammation levels when prasugrel was administrated. It is also interesting to note that in the arthritis-induced animals treated with prasugrel, the plasma levels of IL-10 were significantly decreased. Note: treatment with prasugrel alone did not change the cytokine profile.

**Table 1:** Cytokine profile.

Cytokine	Untreated	Prasugrel	PG-PS	PG-PS+Prasugrel
**IL-1β**	44.66±11.64	30.65±12.86	88.6±23.19	104.1±16.39*
**IL-6**	21.42±2.71	21.96±1.99	142.0±27.59**	119.4±15.11*
**IL-4**	21.81±2.82	14.68±3.91	11.74±1.62	11.05±1.34
**IL-10**	412.4±268.2	391.2±118.9	539.8±221.5	17.81±1.91**,††
**IL-17**	0.99±0.08	0.94±0.09	685.9±301.5**	246.8±50.18*
**PF4**	1.60±0.22	1.09±0.56	1.7±0.38	2.29±0.90**
**MCP-1**	89.72±4.87	83.47±2.79	398.0±79.21**	447.7±69.06**
**TNF α**	17.08±1.87	13.06±0.55	15.24±1.03	13.11±0.55
**RANTES**	12.08±2.13	12.17±2.74	24.54±4.12	28.97±2.77
**MIP1 α**	4.58±0.2	5.08±0.68	10.68±2.47	10.9±1.4*

Plasma cytokine levels in rats following prasugrel administration in PG-PS induced arthritis model.

All values are in pg/mL except for RANTES that are in ng/mL. Values represent the mean ± SEM.

* *p<0.05*,

** *p<0.01* (vs. control groups [untreated group and prasugrel group]).

†† *p<0.01* (vs. PG-PS group).

### Blood Chemistry Profile was not Altered with PG-PS-induction

Serum levels of the blood chemistry profile showed no differences among all groups (data not shown). Even though that the majority of the active metabolite of prasugrel is produced in the intestine, it is important to mention that no changes in hepatic function after PG-PS induction suggest any impairment of the liver to metabolize prasugrel.

## Discussion

The high incidence of cardiovascular disease and the subsequent necessity for coronary intervention have led to a need for safe and effective anti-thrombotic therapies. Among them, clopidogrel has been used as the gold standard treatment to reduce thrombus formation, and recently prasugrel has been added as a therapeutic tool [21]. Previously we reported a detrimental effect of clopidogrel administration in induced-arthritis rats. This study aimed to evaluate the effect of prasugrel in the same erosive arthritis model. Both clopidogrel and prasugrel aggravated the arthritis manifestations and inflammation in these rats, though there were several differences between both studies. We observed that prasugrel aggravated the arthritis even in the acute phase of the disease, as judged by a significant increment in joint diameter of the animals, while clopidogrel did not [15]. Interestingly the ankle inflammation reached the highest grade of severity by physical and/or histopathological parameters in some animals treated with prasugrel. Even though we are aware of the limitation of our comparisons between these two studies since they were not performed side-by-side, these data suggest that prasugrel compared with clopidogrel may affect the joints at different stages of the disease and it may have an impact on the severity of the arthritis.

Platelets and platelet-derived microparticles have been associated with inflammatory processes of autoimmune disorders including rheumatoid arthritis, systemic lupus erythematosus and sjögren syndrome [14], [22]–[27]. The study of the pathogenesis of these platelet-derived microparticles has increased over the last decade, and multiple mechanisms have been proposed [14], [23], [24], [27]. Platelet-derived microparticles have been directly involved with leukocyte activation, monocytes in particular, during inflammation [28]. Prasugrel and clopidogrel are considered selective antagonists of the P2Y_12_ receptor in platelets and inhibit platelet microparticle generation [29]. Yet, studies treating animals with thienopyridine compounds in the erosive arthritis model suggest an effect of the active or inactive metabolites of the drug in cells other than platelets, resulting in exacerbation of the inflammatory response. Indeed Boillard et al [14] found an increment in the diameter of the joints of the mice treated with the arthritogenic K/BxN serum as well, however the purpose of their study was to demonstrate the involvement of platelets in the development of arthritis. To our knowledge, there is no information related to inflammation levels and ankle joint diameter after prasugrel treatment.

IL-10 plasma levels were dramatically decreased in the arthritic prasugrel treated animals. The anti-inflammatory cytokine IL-10 is produced by monocytes and type II T helper cells [30], [31] and inhibits the cellular secretion of TNF-α, IL-1 and IL-6 [32]. Using a murine animal model of arthritis, Kochetkova et al [33] demonstrated a preventive effect against arthritis with overexpression of IL-4, IL-10 and TGF-β with subsequent reduction of IL-17 and IFN-γ. Monocytes produce IL-10 upon activation by cell-to-cell interaction or by response to other interleukins or factors. Wang et al [34] reported mRNA expression of several P2Yreceptors, including P2Y_12_, in monocytes and lymphocytes. The action of the thienopyridine metabolites (active or inactive) may interact with P2Y_12_ or other P2Y receptors present in monocytes or lymphocytes, hence it may inhibit IL-10 synthesis with further exacerbation of the immune response in this animal model. Interestingly, these results are consistent with what previously observed in arthritic animals treated with clopidogrel. In order to evaluate the arthritogenic effect of activated platelets after prasugrel treatment, we investigated plasma levels of PF4, which is an anti-inflammatory chemokine released from the alpha granules of activated platelets [35] and a potent chemo-attractant for neutrophils, monocytes and fibroblasts [35]. In our previous study using the PG-PS-induced erosive arthritis model when clopidogrel administration aggravates arthritis, PF4 plasma levels were elevated in PG-PS-induced animals, however there was no difference with concomitant administration of clopidogrel [15]. In this study, measurements of the PF4 levels showed an increased in PF4 levels in all PG-PS-induced animals and even more with prasugrel, suggesting a possible arthritogenic effect of activated platelets after prasugrel treatment.

Visceral inflammatory manifestations of the PG-PS-induction were studied as well. Formation of granulomas in liver was observed in all animals induced by PG-PS after 21 days, however, prasugrel treatments significantly increased liver inflammation. Granuloma formation is mainly due to macrophage infiltration, although other immune cells such as neutrophils and lymphocytes play a role in the recruitment of monocytes into soft tissues. This increased macrophage infiltration could be platelet-mediated; therefore the decrease in platelet activity could alter the response of other cells of the immune system. Alternatively, since both monocytes and lymphocytes have shown to express P2Y_12_ receptor [36], prasugrel active metabolites might directly target also these cells, and alter monocyte differentiation into macrophages or lymphocyte stimulation. Furthermore, other prasugrel metabolites could interact with immune cells and alter their response to inflammation. Our group have shown that neutrophil activation is inhibited by prasugrel metabolites, despite the fact that these cells did not seem to express P2Y_12_ receptor [37]. This inhibition of neutrophil functions could be responsible for the increased levels of inflammation observed after prasugrel exposure. To our knowledge, the neutrophilia observed in this animal model after prasugrel exposure has not being observed in humans treated with any thienopyridine compound.

In summary, treatment with clopidogrel or prasugrel after induction of arthritis by PG-PS resulted in aggravation of the clinical manifestations of this animal model in the joints, but also in liver damage probably due to increased cell activation. This effect could be platelet-mediated, but as the effects of both clopidogrel and prasugrel active metabolites on platelets are identical, we conclude that the thienopyridines metabolites exert off-target effects on other immune cells to cause aggravated inflammation.
